# Application of Odd Harmonic Resonances of a Single Crystal to Generation and Reception of Superharmonic Waves for Sensitive Monitoring of Heat-Treated Materials

**DOI:** 10.3390/ma16134777

**Published:** 2023-07-02

**Authors:** Hyunjo Jeong

**Affiliations:** Department of Mechanical Engineering, Wonkwang University, Iksan 54538, Republic of Korea; hjjeong@wku.ac.kr

**Keywords:** super harmonic generation, odd harmonic resonance, quintic nonlinearity parameter, source nonlinearity, precipitation heat treatment

## Abstract

In nonlinear ultrasonic testing, the quadratic and more recently cubic nonlinearity parameters are frequently measured as a quantitative indicator of damaged material state. Application of higher-order harmonics can improve the sensitivity of detection and monitoring for damages and microstructures due to their higher values of nonlinearity parameters. The excitation and reception of higher-order harmonics, so-called superharmonics, which use the third to fifth harmonics arising from nonlinear wave propagation, is not sufficiently investigated and applied. The purpose of this communication is to develop a highly sensitive superharmonic nondestructive technique that efficiently generates and receives third- and fifth-order harmonics using the odd harmonic resonances of a single piezoelectric crystal. The method focuses on the measurement of fifth harmonic generation and reception, and the calculation of the relative quintic nonlinearity parameter (δ’). The method also addresses the issue of source nonlinearity that may be contained in the measured fifth harmonic amplitude. The measurement results of δ’ for a series of precipitation heat-treated samples clearly show a much better sensitivity than the results of the cubic nonlinearity parameter (γ’). The proposed method enables a highly sensitive and true pulse-echo mode nonlinear ultrasound testing.

## 1. Introduction

Harmonic generation measurement has been recognized as a promising nonlinear ultrasound testing (NLUT) tool for assessing material conditions or microdamage of various materials [1,2]. Second harmonic generation (SHG) techniques are most widely employed and provide the quadratic nonlinearity parameter (*β*) that is calculated using the received fundamental and second harmonic amplitudes. The cubic nonlinearity parameter (γ) can be obtained similarly by third harmonic generation (THG) techniques, and is often used as a more sensitive parameter in many applications. Compared to β, γ is expected to provide much higher sensitivity and resolution for the same damage because γ has in general a higher value than β. The advantages of measuring γ from nonlinear longitudinal waves were reported in the literature for fatigue cracks [3,4], plastic deformation [5,6,7], microstructures [8,9,10], dislocation [11], and precipitation [12,13] of metals. In addition, third harmonic generation using Lamb waves and Rayleigh surface waves was studied for different application purposes such as the effect of microstructure evolution on higher harmonic generation of guided waves [14], third harmonic Lamb waves for early fatigue damage detection [15], and third harmonic shear horizontal waves for material degradation monitoring [16,17].

Based on these investigations, it is expected that the fourth- or fifth-order (quartic or quintic) nonlinearity parameters will provide superior sensitivity compared to the third-order (cubic) nonlinearity parameter if generation and reception of the fourth or fifth harmonic are possible. However, the excitation and reception of so-called superharmonics, which use third to fifth harmonics generated from nonlinear wave propagation in a target material, have not been sufficiently investigated and applied. The purpose of this short communication is to develop a highly sensitive superharmonic nondestructive testing method that efficiently generates and receives the third and fifth harmonics using the odd harmonic resonances of a single piezoelectric crystal. This article focuses on the fifth harmonic generation and reception, and the calculation of the relative quintic nonlinearity parameter (*δ*’) with source nonlinearity correction.

One of the most important experimental elements in superharmonic generation (SHG) measurement is the transmit/receive transducer. Considering the odd SHG, the third harmonic displacement amplitude is about three orders of magnitude lower than the fundamental amplitude and the fifth harmonic displacement amplitude is about three orders of magnitude lower than the third harmonic amplitude. Therefore, efficient excitation and highly sensitive detection of these odd superharmonics are quite important. Broadband single-element transducers have been used to measure γ of liquids in the through-transmission testing [18]. Separately arranged transmitter/receiver ultrasonic transducers were also used for the detection of third harmonic signal with high selectivity and high reception sensitivity in tissue harmonic imaging [19]. Ultrasonic receivers with double-peak-type frequency characteristics were designed and fabricated for third harmonic reception [20]. The peak on the low-frequency side receives the fundamental wave, and another peak on the high-frequency side receives the third harmonic component. A transmit/receive transducer that is sensitive at both fundamental and third harmonic frequencies was proposed [21]. These frequencies respectively correspond to the fundamental and third harmonic thickness resonances of a piezoelectric transducer element. Thickness resonances of piezoelectric elements are well known, but the properties of higher harmonic thickness resonances are further explored in this article and applied to monitoring the variation of quintic nonlinearity parameter (δ’) in precipitation heat-treated specimens as the true pulse-echo NLUT.

The odd harmonic thickness resonances of a single crystal transducer are very efficient in generating and receiving odd superharmonics. The practical advantage of using odd superharmonics lies in the realization of pulse-echo mode testing, thus having great potential in field applications. Unlike the behavior of the second harmonic in the pulse echo test [22], the behavior of the third and fifth harmonics at the stress-free boundary has no phase difference between the initially generated and the newly generated odd harmonic components after reflection. Therefore, they can be treated as a continuous wave propagating twice the sample thickness, and the nonlinear components accumulate in proportion to the propagation distance.

This article also addresses the problem of source nonlinearity that may be present in the measured fifth harmonic amplitude. In odd SHG measurements, the source nonlinearity is inevitably accompanied, so checking its presence and properly correcting it are very important for the accurate measurement of δ’. A simple and practical method for source nonlinearity correction is proposed in this study. The measurement results of δ’ on a series of precipitation-heat treated samples clearly exhibit much better sensitivity than the results of the cubic nonlinearity parameter (γ’). The proposed method enables highly sensitive and pulse-echo mode nonlinear ultrasound examination of microdamaged materials.

## 2. Basic Theory, Materials, and Nonlinear Ultrasound Testing

This section covers the measurement of the relative quintic nonlinearity parameter (δ’) in the pulse-echo mode for 1 cm thick aluminum specimens whose microstructure changes due to precipitation heat treatment. The relation between δ’ and heat treatment time will be studied. Moreover, it will be demonstrated that a single crystal with odd harmonic resonances can serve as a highly sensitive transducer for excitation and reception of fifth harmonic waves.

The heat treatment of metal alloys results in a change in the microstructure of materials and thus a change in mechanical properties. As a representative example, aluminum alloys are hardened and strengthened by precipitation hardening process through which extremely small, uniformly dispersed particles of a second phase form within the original phase matrix [23]. The fine particles of an impurity phase impede the movement of dislocations and serve to harden the material. Since dislocations are the dominant mechanism of harmonic generation, the fifth harmonic wave will generate different values of the quintic nonlinearity parameter with the evolution of the microstructure.

### 2.1. Theory

Consider one-dimensional plane wave propagation in an isotropic solid with quadratic nonlinearity. The equation of motion governing the longitudinal wave propagation in the *x* direction can be deduced as [24,25].

(1) $$\frac{1}{c^{2}}\frac{\partial^{2}u}{\partial t^{2}}-\frac{\partial^{2}u}{\partial x^{2}}=-\beta \frac{\partial u}{\partial x}\frac{\partial^{2}u}{\partial x^{2}}$$

where u=u(x,t) is the particle displacement at any given position *x* and time *t*, and *c* is the longitudinal wave speed. β is the quadratic nonlinear parameter given by $\beta =3+C_{111}/C_{11}$ where $C_{11}$, and $C_{111}$ are the second-, and third-order elastic constants. In Equation (1), terms of higher-order nonlinearity parameters that involve the fourth- and higher-order elastic constants were neglected.

Assume a harmonic displacement boundary condition $u\left(0,t\right)=U_{0}\cos\left(\omega t\right)$ prescribed at x, where $U_{0}$ is the initial source amplitude, ω is the angular frequency, and *k* is the wave number. Keck and Beyer [26] provided an exact solution to Equation (1) for the particle velocity in terms of Bessel function. Following Breazeale and Ford [27], and Thompson et al. [24], the displacement solution *u* can be determined by making a power series expansion of the exact solution,

$$u=U_{0}\cos\left(kx-\omega t\right)+\frac{\beta U_{0}^{2}k^{2}x}{8}\cos2\left(kx-\omega t\right)+\frac{\beta^{2}U_{0}^{3}k^{4}x^{2}}{32}\cos3\left(kx-\omega t\right)$$

(2) $$+\frac{\beta^{3}U_{0}^{4}k^{6}x^{3}}{96}\cos4\left(kx-\omega t\right)+\frac{\beta^{4}U_{0}^{5}k^{8}x^{4}}{128}\cos5\left(kx-\omega t\right)+.\;.\;.\;$$

Equation (2) provides the displacement solutions for the fundamental and the higher harmonics up to the fifth order generated by the propagating longitudinal waves. The displacement amplitudes of these waves at distance x can be identified as follows: $U_{1}\left(x\right)=U_{0},\;U_{2}\left(x\right)=\beta U_{0}^{2}k^{2}x/8$, $U_{3}\left(x\right)=\beta^{2}U_{0}^{3}k^{4}x^{2}/32$, $U_{4}\left(x\right)=\beta^{3}U_{0}^{4}k^{6}x^{3}/96$, $U_{5}\left(x\right)=\beta^{4}U_{0}^{5}k^{8}x^{4}/128$.

For numerical calculation of harmonic amplitudes, the following acoustic properties are used:

$U_{0}=U_{1}=1\times 10^{-8}\;m$, f=5 MHz, c=6400 m/s, $k=\omega /c=4.91\times 10^{3}$, β=15  [28], x=0.05 m.

Then, the following displacement amplitudes are obtained:

$U_{2}=2.26\times 10^{-10}\;m$, $\;U_{3}=1.02\times 10^{-11}\;m,U_{4}=6.15\times 10^{-13}\;m,U_{5}=8.33\times 10^{-14}\;m.$

The second harmonic amplitude ($U_{2}$) is about two orders of magnitude lower than the fundamental ($U_{1}$). The third harmonic amplitude ($U_{3}$) is about one order of magnitude lower than the second harmonic ($U_{2}$). The fifth harmonic amplitude ($U_{5}$) is about two to three orders of magnitude lower than the third harmonic ($U_{3}$). These results indicate the need for highly sensitive detection of fifth harmonic amplitude.

We focus on the fundamental and odd harmonics, and denote the nonlinearity parameters present in the third- and fifth-order harmonic amplitudes as $\beta^{2}\Rightarrow \gamma$ and $\beta^{4}\Rightarrow \delta$ for convenience. These parameters are called the cubic and quintic nonlinearity parameters, respectively. Then, γ at distance x can be defined from $U_{1}\left(x\right)$ and $U_{3}\left(x\right)$, while δ can be defined from $U_{1}\left(x\right)$ and $U_{5}\left(x\right)$.

(3) $$\gamma \left(x\right)=\frac{32U_{3}\left(x\right)}{k^{4}x^{2}U_{1}^{3}\left(x\right)}\;$$

(4) $$\delta \left(x\right)=\frac{128U_{5}\left(x\right)}{k^{8}x^{4}U_{1}^{5}\left(x\right)}\;$$

These are the plane wave displacement-based “absolute” nonlinearity parameters. The “relative” nonlinearity parameter, γ’ and δ’, can be defined more conveniently by using the spectral peak values of the received electrical signal. If it is not necessary to distinguish the thickness and wave speed of the specimens, they are simply expressed as

(5) $$\gamma ’=\frac{A_{3}}{A_{1}^{3}}\;$$

(6) $$\delta ’=\frac{A_{5}}{A_{1}^{5}}\;$$

where $A_{1}$, $A_{3}$ and $A_{5}$ are the peak values of the magnitude spectrum at the fundamental, third harmonic and fifth harmonic frequencies. It is noticed that the effects of diffracton and attenuation were not taken into account when deriving Equations (3)–(6).

### 2.2. Specimens

**Figure 1:**
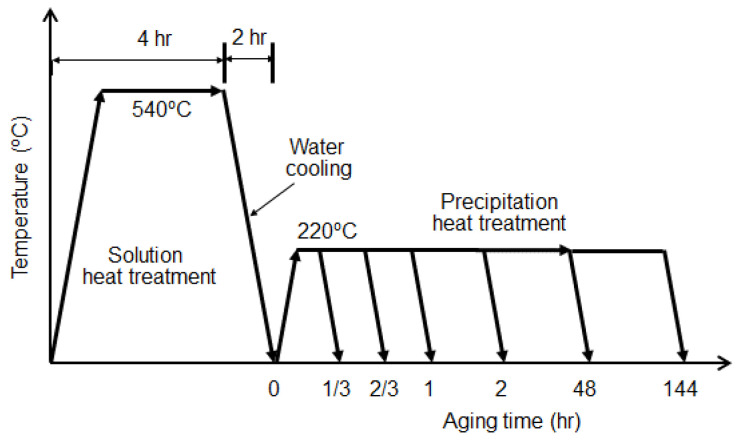
Details of heat treatment for precipitation hardening of aluminum alloys.

In order to conduct the heat treatment, aluminum alloy 6061 specimens with a thickness of 1 cm were prepared. Then, the specimens were subjected to a thermal cycle consisting of solution heat treatment and precipitation heat treatment, as shown in Figure 1. As shown in Figure 1, all the specimens were solution-heat-treated first at 540 °C for 4 h and then cooled in water for 2 h. After water cooling, artificial aging treatment was performed at different times at a temperature of 220 °C. A total of 7 specimens were prepared: one right after water quenching (0 h) and six after artificial aging with different times (1/3, 2/3, 1, 2, 48, and 144 h).

### 2.3. Nonlinear Ultrasound Testing

A finite amplitude, pulse-echo test is conducted for superharmonic generation (SHG) measurement. The transmit and receive transducer (T/R) is a single crystal lithium niobate (LiN) of 5 MHz center frequency and 9.5 mm diameter. The pulse-echo frequency response of this transducer is shown in Figure 3. A series of SHG measurements were performed on the precipitation heat-treated AL samples of various aging times.

**Figure 2:**
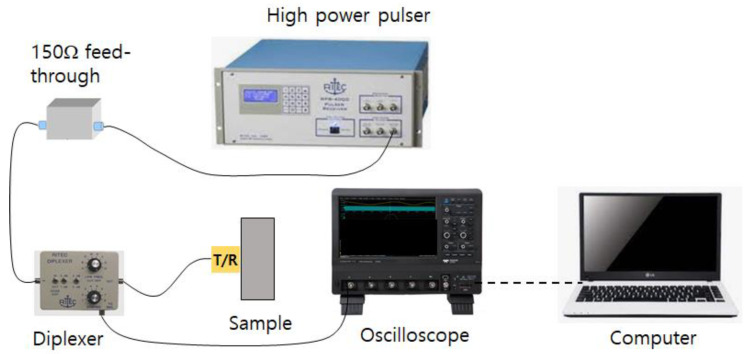
Schematic of the experimental setup for superharmonic generation measurements using the finite amplitude, pulse-echo method.

Figure 2 shows the experimental setup for SHG measurement in the pulse-echo mode. A high power toneburst pulser (RPR-4000, RITEC, Warwick, RI, USA) is used to produce a high voltage 5 cycle toneburst tuned to the fundamental frequency (5 MHz) that is applied to the transmitter via a 150 Ohm high power feedthrough and a broadband diplexer (RDX-6, RITEC, Warwick, RI, USA), a passive device for allowing a single transducer to be used both for transmitting and receiving sound in pulse-echo testing. The receiver signal is captured on a digital storage oscilloscope (WaveSurfer 3024, Teledyne LeCroy, Chestnut Ridge, NY, USA). Seven different input voltages (from 0 to 60 power levels in 10 level step) are applied from the high power pulser. These input power levels correspond to about 30–440 $V_{\mathrm{peak}}\;$ at the transmitter.

## 3. Results and Discussion

### 3.1. Frequency Response of Transmit and Receive Transducer

**Figure 3:**
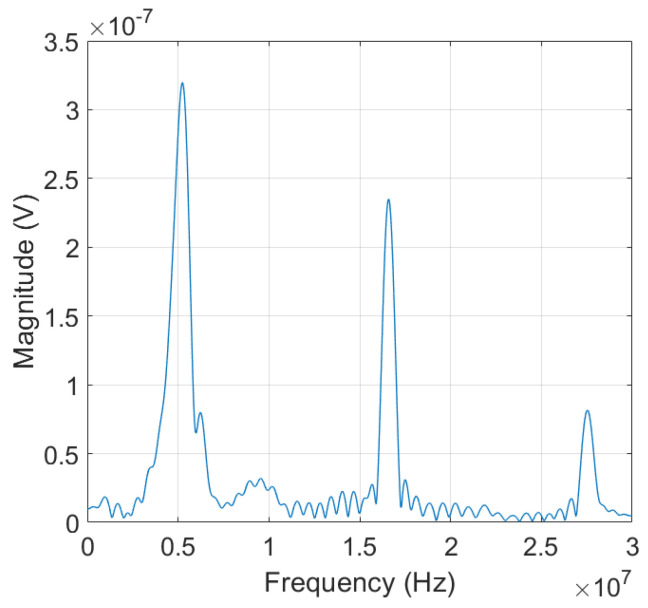
Pulse-echo frequency response of the LiN transducer with 5 MHz nominal center frequency.

Prior to SHG measurement, the frequency response of the LiN single crystal (5 MHz nominal center frequency and 0.5 inch dia.) was measured in the linear ultrasonic range. The experiment was carried out using a broadband pulser and a 1 cm-thick Al block in pulse-echo mode. A negative spike pulse was applied to the transducer to generate a pulsed ultrasound in the Al block. This input pulse was acquired from the Panametrics 5052 pulser/receiver. The frequency spectrum obtained from the pulse-echo test of the Al block is shown in Figure 3. The spectrum shows a peak magnitude at 5 MHz, which is the nominal center frequency of the transmit/receive element or the fundamental resonance peak. The resonance peaks at about 15 MHz and 25 MHz correspond to the 3rd overtone and 5th overtone resonance frequencies, respectively. The peak values at the fundamental and third harmonic frequencies are in the same order of magnitude, and the peak value at the fifth harmonic frequency is about one order of magnitude lower than these. This characteristic frequency spectrum of the transducer will act as a transmitter for effective excitation of the fundamental frequency wave and the generation of odd superharmonics. Also, the transducer can be used very effectively for selective and highly sensitive reception of the fundamental wave and odd superharmonics.

### 3.2. Received Output Signal and Magnitude Spectrum

**Figure 4:**
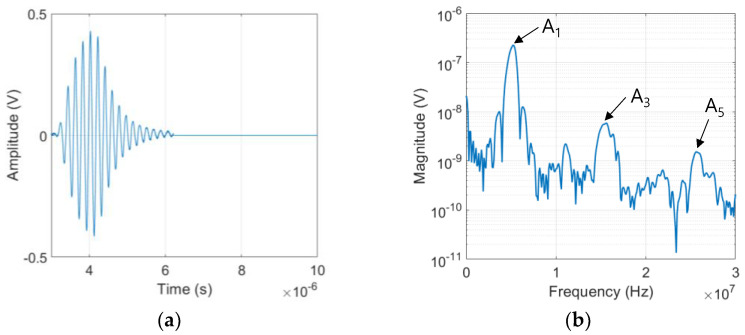
Received output signal: (**a**) typical time domain waveform; and (**b**) magnitude spectrum.

The SHG measurement was performed using the finite amplitude, pulse-echo method from which the peak values of frequency spectrum at the fundamental and odd superharmonic frequencies are obtained to calculate the relative cubic and quintic nonlinearity parameters γ’ and δ’ of each sample. Figure 4a,b shows a typical example of the electrical output signal and its Fourier spectrum acquired from the #1 sample at the input power level 40 when 5 cycles of sine wave toneburst was applied. As shown in Figure 4b, in addition to the fundamental component $A_{1}$ at *f* = 5 MHz, the third and fifth harmonic components $A_{3}\;\mathrm{and}\;A_{5}$ are clearly seen at about 3*f* = 15 MHz and 5*f* = 25 MHz, respectively. Because the LiN element was not tuned and used as fabricated, the superharmonic peaks do not occur precisely at 15 MHz and 25 MHz. It is also noted that the generated third harmonic peak is about 40 dB lower than that of the fundamental wave and the generated fifth harmonic peak is about 20 dB lower than that of the third harmonic. Because of the destructive interference of the two second harmonic components after reflection at the stress-free boundary, the received second harmonic is negligibly small, and the pulse-echo method cannot be used for the reliable measurement of β’. The peak values of the fundamental and superharmonics in the frequency spectrum of the received signal always depend on the input voltage level.

### 3.3. Uncorrected δ’

**Figure 5:**
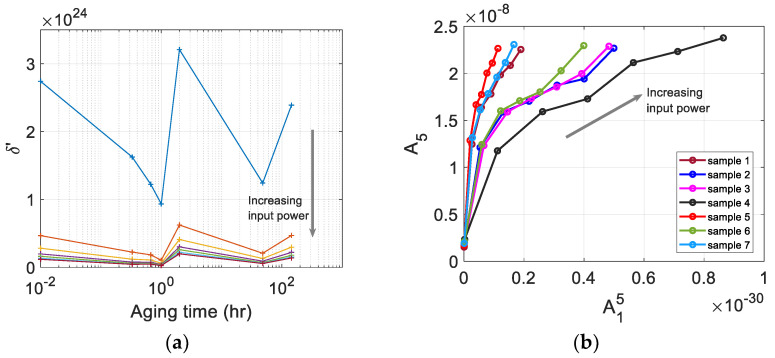
(**a**) The uncorrected quintic nonlinearity parameter δ’, and (**b**) the plot of $A_{1}^{5}$ vs. $A_{5}$ of seven samples measured at seven different input power levels.

The relative quintic nonlinearity parameter of each sample at a specific input power level was calculated using $\delta^{\prime }=A_{5}/A_{1}^{5}$, where $A_{1}$ and $A_{5}$ are the spectral peak values at the fundamental and fifth harmonic frequencies, respectively. Figure 5a shows δ’ of seven samples measured at seven different input power levels. The calculation of these parameters is based on the relationship between $A_{1}^{5}$ and $A_{5}$ measured at seven different input power levels shown in Figure 5b. The results of $\delta^{\prime }$ in Figure 5a are before the source nonlinearity correction was made. A detailed procedure of source nonlinearity correction will be described later, and the relationship between the source nonlinearity corrected $\delta^{\prime }$ and the aging time will also be discussed.

To investigate the dependence of experimentally measured amplitudes on the input power level, Equation (4) is rearranged in a slightly different form using the ratio $U_{5}\left(x\right)/U_{1}^{5}\left(x\right)$, which will depend on the input power level used in the experiment

(7) $${\frac{U_{5}\left(x\right)}{U_{1}^{5}\left(x\right)}|}_{\begin{array}{c} input\\ power \end{array}}=\frac{\delta k^{8}x^{4}}{128}\;$$

Equation (7) indicates that a plot of $U_{1}^{5}$ vs. $U_{5}$ obtained experimentally at different input power levels should follow a straight line with zero y-intercept. However, the actual plot of $A_{1}^{5}$ vs. $A_{5}$ may not be linear across all input power levels used, as shown in Figure 5b. Furthermore, the y-intercept of the fitted line may not pass through the origin. The y-intercept above the origin indicates the amount of source nonlinearity included in the measurement system or the noise floor of the measurement system. Plot of Figure 5b tells us that a proper selection of data range is necessary to be used in the linear fitting process to find the y-intercept. In addition, a plot of initial δ’ at different input power levels similar to Figure 5a is also helpful in selecting an appropriate range of input power level to be used in the fitting process.

### 3.4. Source Nonlinearity Correction

The relation between $A_{1}^{5}$ and $A_{5}$ measured at different input power levels can be ploteed to check the existence of noise floor and/or source nonlinearity in the measured $A_{5}$. Figure 5b shows plots of $A_{1}^{5}$ vs. $A_{5}$ for all seven samples at seven input power levels used.

Looking at Figure 5b, the plot of $A_{1}^{5}$ vs. $A_{5}$ can be divided into two regions according to their slopes as the input voltage increases: a steep slope region at low input power levels below PL20, and a moderate slope region at high input power levels above PL20. There is no strict criterion for selecting a suitable input power range to be used for the linearization process, but in this study, the data from the second region were used for linear fitting to obtain the slope and y-intercept. Since the samples are not thick and the effects of diffraction and attenuation corrections are insignificant [29], only source nonlinearity corrections were taken into account here.

**Figure 6:**
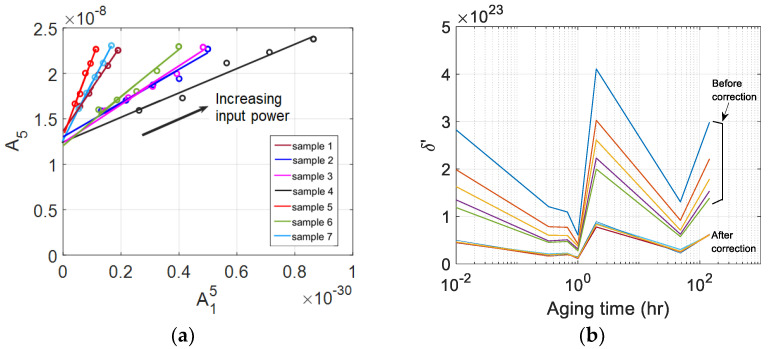
(**a**) The best fit straight line for the plot of $A_{1}^{5}$ vs. $A_{5}$ measured at five input power levels; and (**b**) the calculated δ’ of seven samples after source nonlinearity correction.

The raw data used in the curve fitting and the results of the best fit straight line are shown in Figure 6a. There exists a good linearity between these data in each sample. The y-intercept in Figure 6a is well above the origin, indicating that a significant amount of source nonlinearity is contained in $A_{5}$. The y-intercept of each sample was subtracted from $A_{5}$ of that sample, and the δ’ after source nonlinearity correction was calculated. The results are shown in Figure 6b.

Comparing the results of δ’ before and after source nonlinearity correction (Figure 5a and Figure 6b), the δ’ before correction is widely scattered over the five input power levels used for linear curve fitting, whereas the δ’ after correction is very narrowly clustered. The dependence of the corrected δ’ of each sample on the input power is now greatly reduced, and this decrease in input voltage dependence is more evident at relatively low input voltages. In addition, the magnitude of δ’ decreased and the characteristic behavior as a function of the aging time was maintained after corrections for the source nonlinearity, so the correction appears to have been made adequately in the right direction.

Figure 6b shows the variation of corrected δ’ as a function of aging time. Right after solution heat treatment and water quenching at 220 °C, δ’ shows a continuous decrease until 20 min. of aging time and then a slight increase at 40 min. and a decrease again to reach a minimum at about one hours. After reaching the minimum value, it increases rapidly at about 2 h, then rapidly decreases at 48 h, and gradually increases at 144 h. This behavior of δ’ agrees very well with that of the absolute β measurement results [30], but their sensitivity is different as compared below. The variation of δ’ as a function of the aging time can be explained by the microstructural change of the material due to the generation, evolution, and extinction of the precipitates inside the specimen caused by the precipitation heat treatment and aging time [31,32].

### 3.5. Average δ’ after Source Nonlinearity Correction and Comparison of Sensitivity

**Figure 7:**
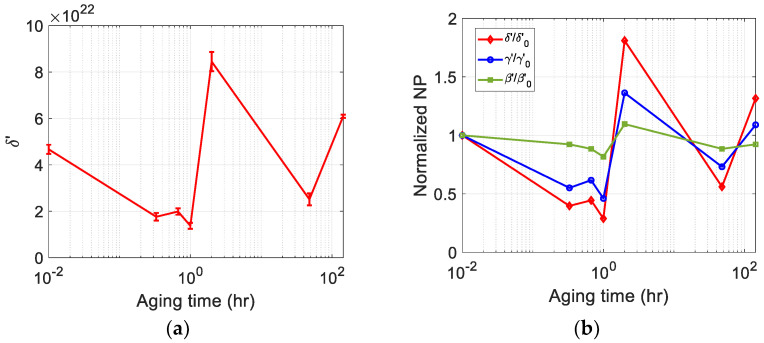
(**a**) The average δ’ after source nonlinearity correction; and (**b**) Comparison of sensitivity using normalized nonlinearity parameters.

The average value of δ’ of each sample was obtained from the source nonlinearity- corrected δ’ of Figure 6b measured at five input power levels from PL20 to PL60. The results are shown in Figure 7a. The error bars here represent the standard deviation of δ’ measured at five input power levels. The maximum error is about 10% that occurs in the sample of 48 h of aging time. Figure 6b shows the widely scattered δ’ before source nonlinearity correction and the strong dependence of δ’ on the input power level. The small error bars after source nonlinearity correction indicate that the source nonlinearity correction greatly reduces the dependence of δ’ on input voltage.

The absolute quadratic nonlinearity parameter, β, was measured in the previous study for the same heat-treated samples using a dual element transducer and the pulse-echo method [30]. In this section, the normalized δ’ after source nonlinearity correction was compared with the normalized β. In addition, the normalized δ’ was also compared with the normalized γ’, the relative cubic nonlinearity parameter measured in the previous study for the same heat-treated samples using the same transmit/receive transducer [33]. The average value of δ’ obtained from the five input power levels shown in Figure 7a was used here as the δ’ of each sample. The values of #1 sample were used for normalization in all three comparisons. The comparison results are shown in Figure 7b. The three normalized nonlinearity parameters β, γ’ and δ’ show a very similar behavior in that the minimum occurs after one hour and the maximum occurs after two hours of aging time.

If we compare the sensitivity, the normalized δ’ shows better sensitivity than the normalized β and γ’ over the entire aging times. In particular, the normalized δ’ at the aging times of one hour and two hours, where all three normalized parameters have minimum and maximum values, shows much better sensitivity than the normalized β and γ’. Since the sensitivity comparison here is not a comparison between absolute nonlinearity parameters, a quantitative sensitivity comparison is difficult. However, qualitatively speaking, the excellent sensitivity of δ’ shown here was attributed to the high sensitivity in the generation and reception of odd superharmonics with the help of of LiN single crystal transducer.

In NLUT, it is basically needed to check the source nonlinearity that may be present in the received harmonic signal. When a piezoelectric transducer is used to generate odd superharmonics, such as the third and fifth order, it is essential to check and properly remove the source nonlinearity since some degree of source nonlinearity is unavoidable [29]. The source nonlinearity elimination method proposed in NLUT and harmonic imaging is a metamaterial filter [34,35] and a harmonic cancellation method [36]. These methods require additional hardware attached to the experimental setup. Compared to these methods, the source nonlinearity correction method used in this study is convenient to apply and has obvious advantages. However, selection of an appropriate input power range for linear curve fitting is very important as it directly affects the resulting value of δ’. It seems necessary to develop a more systematic method for this.

Single crystal piezoelectric elements or transducers that exhibit good characteristics of odd harmonic thickness resonances (f, 3f, 5f, . . . ) can be very effective in exciting and generating the fundamental and odd superharmonic waves (f, 3f, 5f) under the finite amplitude excitation of the toneburst fundamental wave. Their sharp, narrow bandwidth at these frequencies also make them very effective in receiving these waves with good selectivity and high sensitivity. Furthermore, the biggest attraction is the realization of the true pulse-echo mode testing by having these transmission/reception characteristics in one transducer.

The excellent superharmonic properties and pulse-echo testing capability are expected to promote the development of new measurement systems in nonlinear ultrasonic testing and expand field applications. The key content will be the detection, monitoring and imaging of the material state and the evolution of microstructures through superharmonic generation and measurement of cubic and quintic nonlinearity parameters in damaged materials and structures.

## 4. Conclusions

In this article, we have shown that a single element transducer with odd harmonic thickness resonances is very effective in generating and receiving fundamental and superharmonic waves under the finite amplitude excitation of toneburst fundamental wave. The pulse-echo testing could be realized due to the excellent transmit/receive characteristics of the transducer. Superharmonic generation measurements were conducted in the pulse-echo mode to determine the quintic nonlinearity parameter (δ’) for a series of precipitation heat-treated samples with different aging times. The measurement results showed a change in δ’ consistent with the change in microstructure due to the transition of the precipitate at a specific aging time. These δ’ results were in good agreement with those of absolute β and relative γ’, but with much better sensitivity. The sensitivity of a transmit/receive transducer that generates and receives the fundamental wave and superharmonics depends on the transducer’s frequency bandwidth and its strength. Because odd harmonic resonant transducers operate very sensitively around these frequencies as fabricated, they can easily meet frequency bandwidth requirements as sensitive transmit/receive transducers at superharmonic frequencies.

The source nonlinearity problem was addressed based on the assumed linear relationship between $A_{1}^{5}$ and $A_{5}$ where $A_{1}$ and $A_{5}$ are the resulting fundamental and fifth harmonic spectral peaks measured at varying input power levels. The amount of source nonlinearity included in the measued $A_{5}$ could be determined from the y-intercept of the linear cuve fit. The source nonlinearity correction greatly reduced the dependence of δ’ on input voltage and provided an average δ’ with less than 10% error. The source nonlinearity correction method used in this study is convenient to apply and has obvious advantages compared to metamaterial filters and harmonic cancellation methods.

## Data Availability

The data presented in this study are available on request from the corresponding author.
